# The association of workforce configurations with length of stay and charges in hospitalized patients with congestive heart failure

**DOI:** 10.3389/frhs.2024.1411409

**Published:** 2024-12-23

**Authors:** Tremaine B. Williams, Alisha Crump, Pearman Parker, Maryam Y. Garza, Emel Seker, Taren Massey Swindle, Taiquitha Robins, Adrian Price, Kevin Wayne Sexton

**Affiliations:** ^1^ Department of Biomedical Informatics, University of Arkansas for Medical Sciences, Little Rock, AR, United States; ^2^ Department of Epidemiology, University of Arkansas for Medical Sciences, Little Rock, AR, United States; ^3^ College of Nursing, University of Arkansas for Medical Sciences, Little Rock, AR, United States; ^4^ Department of Family and Preventive Medicine, University of Arkansas for Medical Sciences, Little Rock, AR, United States; ^5^ Risc.ai, Little Rock, AR, United States; ^6^ Department of Surgery, University of Arkansas for Medical Sciences, Little Rock, AR, United States; ^7^ Department of Health Policy and Management, University of Arkansas for Medical Sciences, Little Rock, AR, United States

**Keywords:** care delivery, care team composition, congestive heart failure, elixhauser index, risk stratification, electronic health record

## Abstract

**Introduction:**

Clinicians are the conduits of high-quality care delivery. Clinicians have driven advancements in pharmacotherapeutics, devices, and related interventions and improved morbidity and mortality in patients with congestive heart failure over the past decade. Yet, the management of congestive heart failure has become extraordinarily complex and has fueled recommendations from the American Heart Association and the American College of Cardiology to optimize the composition of the care team to reduce the health, economic, and the health system burden of high lengths of stay and hospital charges. Therefore, the purpose of this study was to identify the extent to which specific care team configurations were associated with high length of stay and high-charge hospitalizations of patients with congestive heart failure.

**Methods:**

This study performed a retrospective analysis of data extracted from the electronic health records of 3,099 patients and their hospitalizations from the Arkansas Clinical Data Repository. The data was analyzed using binomial logistic regression in which adjusted odds ratios reflected the association of specific care team configurations (i.e., combination of care roles) with length of stay and hospital charges.

**Results:**

Team configurations that included a nurse practitioner, registered nurse, care manager, and social worker were generally above the median length of stay and median charges when compared to team configurations that did not collectively include all of these roles. Patients with larger configurations (i.e., four or more different care roles) had higher length of stays and charges than smaller configurations (i.e., two to three different care roles). The results also validated the Van Walraven Elixhauser Comorbidity Score by finding that its quartiles were associated with length of stay and charges, an indicator of care demand based on patient morbidity.

**Conclusions:**

Cardiologists, alone, cannot shoulder the burden of improving patient outcomes. Care team configuration data within electronic health record systems of hospitals could be an effective method of isolating and tracking high-risk patients. Registered nurses may be particularly effective in advancing real-time risk stratification by applying the Van Walraven Elixhauser Comorbidity Score at the point of care, improving the ability of health systems to match care demand with workforce availability.

## 1 Introduction

Heart disease is the leading cause of death in the United States with recent counts at 702,880 deaths (0.2% of the US population), annually (1), a precursor to congestive heart failure (CHF) (2, 3). Approximately 6.2 million adults (1.8% of the US population) in the United States have CHF (2). Furthermore, the care outcomes of patients with CHF have varied significantly with heart failure having an annual estimated economic cost of $69 billion by 2030 (4), which has risen substantially from $30.7 billion in 2012 (2). Hospital charges have also ranged from $908 to $84,434 per hospitalization and length of hospital stay ranging from zero to more than 30 days (5–7). Patients with CHF have also been found to be 16% more likely to have a high-charge hospitalization (i.e., the United States dollar amount billed to the payor by the hospital for medical care that is above the median charges for the hospital) when compared to patients with other chronic conditions (8). Correspondingly, advances in pharmacotherapeutics, devices, and related interventions have significantly improved morbidity and mortality; yet, CHF management has become extraordinarily complex over the past decade (3). Therefore, the 2022 Clinical Practice Guidelines for the Management of Heart Failure, developed by the Joint Committee of the American College of Cardiology and the American Heart Association, have recommended advancements in nonpharmacological interventions to work in parallel to improve patient outcomes (3). Specifically, the Joint Committee has recommended that patients receive care from multidisciplinary care teams to optimize the implementation of evidence-based and guideline-directed medical therapy which includes clinical evaluation, diagnoses, and procedural treatments (3). Prior evidence has established that cardiologists, CHF nurses, and other CHF specialists are significantly associated with positive care outcomes (3). However, little evidence exists as to the specific combinations of generalist clinicians within multidisciplinary inpatient teams that are associated with positive outcomes of patients with CHF during care delivery.

The complexity of CHF management requires various types of care team members to engage in care delivery. Management of each inpatient's CHF case is determined by patient-specific criteria (e.g., individual goals for care, socioeconomic and resource access, health literacy, and network of support) (9), requiring diversity in the types of training needed to provide care. The diversity within the care team ensures that gaps in addressing patient needs are minimized, and is most effective when overlapping responsibilities of care roles are diminished (10). At their core, the care teams of hospitalized patients with CHF include but are not limited to various general and specialty physicians (i.e., overall care responsibility, final decision-maker), nurse practitioners (i.e., make diagnosis, optimize medication, telemonitoring, CHF education), registered nurses (i.e., education related to their personal health and condition; and the management of fluid intake), pharmacists (i.e., guideline-directed medical therapy medication selection and dosing), dieticians (i.e., provide dietary education on sodium intake and potassium enrichment), care managers (i.e., post-discharge calls and care coordination), and social workers (i.e., support financial and social needs related to care) (10). Yet, the American College of Cardiology and American Heart Association have recommended investigating the configuration of care teams to reduce hospitalizations and related charges (10–13).

Recommendations from the American College of Cardiology and the American Heart Association have motivated recent investigations regarding the variation in the outcomes of patients with CHF, much of which have suggested that standardization and optimization of the care team can reduce variation and improve patient outcomes. Some investigations have relied heavily on real-world data extracted from electronic health records, which is ideal for addressing the real-world impact of CHF (8). For example, charges have been found to increase with the treatment experience levels among care teams (5). Specific types of care roles engaging in care delivery hospitalizations of patients with CHF have been associated with improved outcomes. For example, patients with the severest cases of CHF (i.e., left ventricular ejection fraction of less than 40%) who had a registered nurse on their care delivery team during a hospitalization were 88% less likely to have a subsequent hospitalization over the seven-year study period when compared to those patients without a registered nurse on their care delivery team (14). However, patients with other care roles providing care during an hospitalization were more likely to be hospitalized: a physician (i.e., 2.97 times more likely) and a care manager (i.e., 119.09 times more likely) when compared to those patients without a physician or care manager on their care delivery team, respectively (14). patients with CHF who had a social worker on their care delivery team were 3.32 times more likely to have a high-charge hospitalization when compared to those patients without a social worker on their care delivery team (14). Patients with a nurse practitioner on their care team were found to significantly reduce the hospitalization charges when compared to those patients without a nurse practitioner on their care delivery team (15, 16). Other studies demonstrated that patients with nurse practitioners on their care team had 9% lower charges than patients with physicians (17). Yet, these findings were limited to understanding the effect of a patient having a single care role such as a registered nurse on their team. Previous studies have excluded combinations of care roles and their association with length of stay and charges. Registered nurses, for example, do not provide medical care to patients with CHF, alone. Therefore, the impact of a registered nurse working with a physician on the length of stay and charges of a patient with CHF, for example, is unknown and provides a gap in understanding how to configure and optimize the inpatient care team.

Before reconfiguration can occur, an evidence base must be established regarding care team configurations that are related to length of stay and charges. An analysis of the combinations of care roles provides a more comprehensive indication of care team configuration influences on length of stay and hospitalization charges. Further evidence will provide information to support optimizing the composition of care teams during hospitalizations, potentially reducing length of stay and charges for patients with CHF. Therefore, the purpose of this study was to identify the extent to which care team configurations of non-specialty clinicians were associated with high length of stay and high charges in patients with CHF.

## 2 Methods

### 2.1 Study design

A retrospective analysis of deidentified electronic health record data was performed (18). The data was analyzed using binomial logistic regression. Odds ratios were used to illustrate the associations between predictor variables (i.e., care team configurations) and outcome variables (i.e., a high length of stay and a high-charge hospitalization). The study procedures (Protocol #26259) were reviewed and approved by the Institutional Review Board at the University of Arkansas for Medical Sciences.

### 2.2 Study setting

The study was conducted at the University of Arkansas for Medical Sciences' main campus in Little Rock, AR, which is the only academic health center in the state. The hospital has 535 beds (i.e., 431 adult beds, 64 bassinets, and 40 psychiatry beds), which generated the study data. All available data on hospitalizations between January 1, 2016, and December 31, 2021 [before CHF guidelines (3) were modified in 2022] were analyzed in SPSS Version 29.

### 2.3 Participants

For patient criteria, data on patient participants were only included if they had at least one heart failure hospitalization during the study period. A hospitalization was defined as an official decision by a licensed clinician to admit a CHF patient for treatment or observation and an assignment to a hospital bed. All non-hospitalization encounters such as primary care visits were excluded from the analysis because of the study purpose. Hospitalizations due to reasons other than heart failure were excluded. Inclusion criteria limited subjects to patients with CHF who were aged 18–89 and had complete data available for analysis after addressing the missingness of study variables. Only one hospitalization per patient within the study period was randomly selected for analysis using a random number generator. The analysis was limited to only one hospitalization per patient because patients generally had multiple hospitalizations, each of which had a different team configuration and violated the mutual exclusivity assumption of the logistic regression by having the same patient within both groups of the dependent variable (19). For example, in charges, the same patient would have had a low-charge hospitalization (i.e., the United States dollar amount billed to the payor by the hospital for medical care that is below the median charges for the hospital) and a high-charge hospitalization for two different hospitalizations (8).

**Figure 1:**
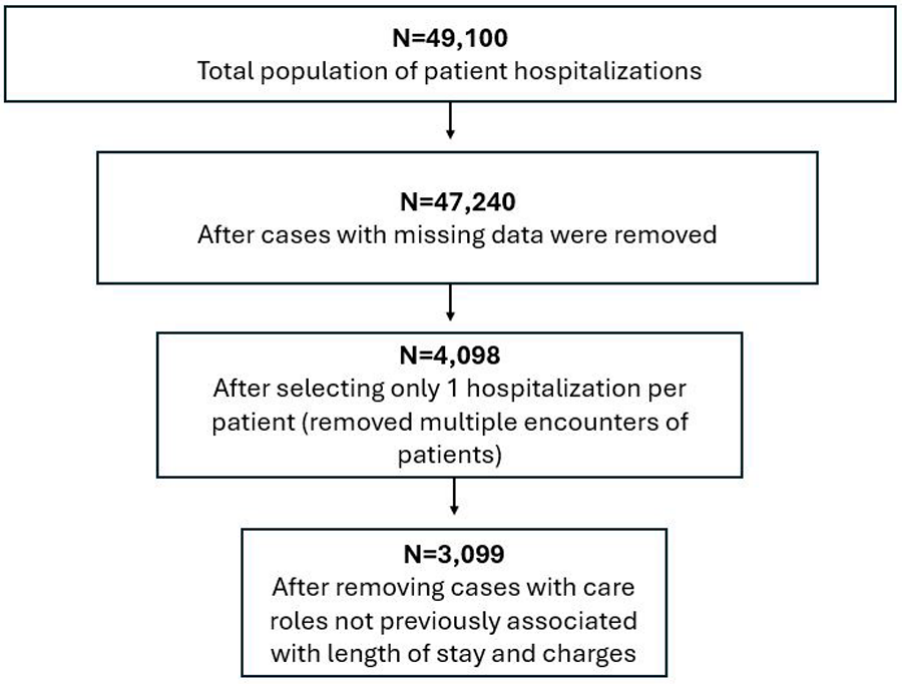
Flow diagram of sample size.

For care team criteria, only patient hospitalizations with the following care roles were included in the analysis: physician, resident, nurse practitioner, registered nurse, care manager, and social worker. Hospitalizations that included other care roles (e.g., pharmacy technician, occupational therapist, medical assistant) were excluded because they had not been previously associated with length of stay and charges (8, 14), or they were less than 5% of the total clinician population in the dataset. Hospitalizations were also excluded if they included more than one type of care role (e.g., two or more nurses) within a single hospitalization to minimize potential confounding caused by the influence of multiple care team members of a specific role. Figure 1 illustrates how the sample size was influenced by the inclusion and exclusion criteria above.

### 2.4 Variables

Demographic study variables of patients were a pseudonym patient ID, age, sex, race, ethnicity, and clinical diagnosis and were collected by care teams during the patient's hospitalization.

Predictor variables were the combinations of care roles on each patient's care team during hospitalization. This data included the following variables: a pseudonym clinician ID and care role (e.g., registered nurse, social worker, etc.).

There were two variables used to address the potential effects of any confounding on the associations between the care team configurations and the outcome variables: left ventricle ejection fraction rates and the Van Walraven Elixhauser Comorbidity Score (VWECS). The VWECS is a single numeric score representing overall disease burden and related in-hospital mortality risk based on International Classification of Disease Version 10 Clinical Modification (ICD-10-CM) codes, which is the standard for recognizing the presence or absence of a disease or condition in electronic health records (20). Ejection fraction rates, captured via transthoracic echocardiogram during hospitalization, were used as a specific indication of heart function and reflected the percentage of the total blood in the heart that is pumped out (21, 22).

Outcome variables were length of stay (days) and hospitalization charges (i.e., the United States dollar amount billed to the payor by the hospital for the medical care that was provided). A complete list of hospital charges is publicly available (23).

### 2.5 Data sources

A dataset was provided from the Arkansas Clinical Data Repository to the study team via a data request. The dataset provided all data for study variables, including an edge list which linked patients with each clinician who provided care during their hospitalization.

### 2.6 Quantitative Variable and transformation

Three continuous variables (age, length of stay, and charges) were dichotomized at the median to establish a high/low threshold to meet the assumption of dichotomous variables in logistic regression (Table 1). For example, a “high length of stay” was defined as a number of days above the median number of days for a hospitalization and was transformed to “1”, reflecting “a high length of stay”. Any number of days below the median was transformed to “0”, reflecting “a low length of stay/not a high length of stay”. Therefore, all variables in the study were dichotomous when analyzed in the logic regression.

**Table 1:** Variables, sources, and transformations.

Original variable	Original value range	Transformed variable name	Transformed variable definition	Value
Age	18–89	High Age	Age above the median	1
Age	18–89	Low Age	Age below the median	0
Length of stay	>0 days	High length of stay	A length of stay above the median	1
Length of stay	>0 days	Low length of stay	A length of stay below the median	0
Charges	≥$0 dollars	High-charge hospitalization	A US dollar amount billed to the payor by the hospital for medical care that was above the median charges for the sample	1
Charges	≥$0 dollars	Low-charge hospitalization	A US dollar amount billed to the payor by the hospital for medical care that was below the median charges for the sample	0
Ejection fraction	15–100%	HFrEF	A patient with ≤40% of the total blood in the heart that is pumped out	1
Ejection fraction	15–100%	HFmrEF	A patient with ≥41%–49% of the total blood in the heart that is pumped out	1
Ejection fraction	15–100%	HFpEF	A patient with ≥50% of the total blood in the heart that is pumped out	1
VWECS	−19 to 89	Low mortality risk	A patient with a VWECS of ranging from −19 to 7)	1
VWECS	−19 to 89	Medium mortality risk	A patient with a VWECS of ranging from 8 to 34)	1
VWECS	−19 to 89	High mortality risk	A patient with a VWECS of ranging from 35 to 61)	1
VWECS	−19 to 89	Very high mortality risk	A patient with a VWECS of ranging from 62 to 89)	1

While more than one member of a specific care role providing care for a patient during their hospitalization could have been a pseudo-indicator of poor patient health, we utilized the VWECS and ejection fraction rates as more objective measures of CHF severity and overall health severity. As a specific measure of heart failure severity, three dichotomous variables were created based on ejection fraction rates consistent with the New York Heart Association Classification and practice standards: reduced ejection fraction (i.e., “HFrEF”; ≤40%), mildly reduced ejection fraction (i.e., “HFmrEF”; ≥41%–49%), and preserved ejection fraction (i.e., “HFpEF”; ≥50%) (21, 22). More broadly, as an indicator of overall health severity, four dichotomous variables were created to group patients with similar mortality risk using each patient's VWECS. The VWECS was chosen because it is a widely used and validated predictor of length of stay and charges. The VWECS compounds 21 conditions (e.g., CHF, diabetes, hypertension, etc.) into a single numeric score, and each condition is associated with a specific weighted numerical value between −7 and 12 (19, 24). The overall score ranges from −19 to 89 (20, 24). The individual numerical values associated with each specific patient's set of conditions is calculated by totaling the sum of the weights. The four dichotomous variables were created by segmenting the VWECS into equal quartiles, representing elevated ranges of mortality risk (20, 24). The quartiles were categorized as low mortality risk (i.e., a VWECS of ranging from −19 to 7), medium mortality risk (i.e., a VWECS of ranging from 8 to 34), high mortality risk (i.e., a VWECS of ranging from 35 to 61), and very high mortality risk (i.e., a VWECS of ranging from 62 to 89).

### 2.7 Statistical analysis

Odds ratios were adjusted for the effects of age, sex, race, ethnicity, health failure severity, and overall health severity, based on the likelihood that participants had a high length of stay and a high-charge hospitalization. Furthermore, adjusted odds ratios provide the associations that care team configurations had with high length of stay and high-charge hospitalization. A chi-squared omnibus test of model coefficients was used to determine if the model with care team configuration included as a predictor was an improvement in fit over the baseline models with no predictor (*p* < 0.05).

## 3 Results

### 3.1 Participant demographics

The study identified an overall sample of 3,099 patients with CHF who were provided care during the study period. Table 2, below, provides demographic information on the 3,099 patients and their hospitalizations. Caucasian Americans accounted for 64% (*n* = 1,978) of all patients and hospitalizations, with Native Hawaiian and other Pacific Islanders accounting for the smallest racial group at less than 1% (*n* = 1) of patients and hospitalizations. Approximately 2% of patients (*n* = 49) were of Hispanic, Latin, and Spanish ethnicity. Of the 3,099 total patients, 28% of patients (*n* = 856) had an ejection fraction of less than or equal to 40%, HFrEF, the severest cases of heart failure diagnosis. Twelve percent of patients (*n* = 377) had an ejection fraction greater than or equal to 41% but less than or equal to 49%, formally referred to as mildly reduced ejection fraction (i.e., HFmrEF), representing intermediate cases of heart failure diagnosis. Sixty percent of patients (*n* = 1,866) had an ejection fraction greater than or equal to 50%, formally referred to as preserved ejection fraction (i.e., HFpEF), the least severe cases of heart failure diagnosis. The logistic regression models in Tables 5 and 6 were evaluated using a chi-squared omnibus test of model coefficients which determined they were an improvement in fit over the baseline models with no predictors (*p* < 0.05).

**Table 2:** Overall patient demographics (*N* = 3,099).

Demographic variables	Patients (*N* = 3,099)
Median age	68
Race, number (%)	
Caucasian American	1,978 (64%)
African American	1,011 (33%)
Asian American	15 (<1%)
American Indian/Alaskan Native	8 (<1%)
Native Hawaiian/Other Pacific Islander	1 (<1%)
Other race	86 (3%)
Ethnicity, Hispanic/Latin/Spanish	49 (2%)
Sex, number (%)	
Male	1,576 (51%)
Female	1,523 (49%)
Heart Failure Severity: Ejection Fraction	
Reduced ejection fraction (≤ 40%)	856 (28%)
Mildly reduced ejection fraction (≥ 41%–49%)	377 (12%)
Preserved ejection fraction (≥ 50%)	1,866 (60%)
Overall Health Severity: Van Walraven Elixhauser Comorbidity Score	
Low Mortality Risk (−19 to 7)	580 (19%)
Medium Mortality Risk (8–34)	2,316 (75%)
High Mortality Risk (35–61)	201 (6%)
Very High Mortality Risk (62–89)	2 (<1%)

During the 3,099 hospitalizations (i.e., one patient per hospitalization), the 3,099 patients were provided care by 10,272 healthcare professionals. Table 3 provides demographic and descriptive information on the number and types of healthcare professionals who provided care and the number of times each type of healthcare professional was present during all hospitalizations. At least 1 registered nurse provided care to all 3,099 patients and was the largest care role engaged in care delivery. Social workers were the smallest number of care roles by group and composed 9% of total clinicians (*n* = 887).

**Table 3:** Number and percentage of care roles found in hospitalizations.

Care roles	Number of clinicians (%) (*N* = 10,272)
Physician	2,141 (21%)
Resident	1,515 (15%)
Nurse practitioner	1,125 (11%)
Registered nurse	3,099 (30%)
Care manager	1,505 (14%)
Social worker	887 (9%)

Based on the six care roles in Table 3, there were a total of 720 possible combinations of team configurations that could have been found within the 3,099 hospitalizations. The figure demonstrates that registered nurses were centrally connected to all providers (i.e., physicians, residents, and nurse practitioners). Additionally, it shows that care managers and social workers were mostly engaged in care delivery hospitalizations that also included physicians.

Table 4 provides demographic and descriptive information on the 28 care team configurations found within all 3,099 hospitalizations. These models were composed of the specific combinations of the individual clinicians who provided care (Table 3). The largest number of patients were found to only have the combination of a physician and a registered nurse providing care during a hospitalization as indicated by 389 patients with a “P + RN” team configuration in Table 4. The smallest number of patients was found in two models: the combination of a resident, nurse practitioner, registered nurse, care manager, social worker, and the combination of nurse practitioner, registered nurse, care manager, social worker, as indicated by 29 patients with an “R + NP + RN + CM + SW” and a “NP + RN + CM + SW” team configurations in Table 4. The largest length of stay (i.e., 3.66 days) was found in patients with a team configuration of “NP + RN + CM + SW” while the smallest (i.e., 2.27 days) was found in patients with an “R + RN” team configuration. The largest dollar amount of hospital charges (i.e., $91,208) was found in patients with a team configuration of “P + NP + RN + CM + SW” while the smallest (i.e., $49,759) was in patients with an “R + RN” team configuration.

**Table 4:** Care team configuration (*n* = 3,099) demographics stratified by patient care teams.

Care team configuration	Number of patients	LOS		Charges	
		Median	Range	Median	Range
Overall (All Models)	3,099	1	3.66	$91,208	$91,208
P + R + NP + RN + CM + SW	58	3.67	1.58	$91,208	$61,052
P + R + NP + RN + CM	70	3.67	2.63	$91,208	$64,380
P + R + NP + RN + SW	56	3.67	2.75	$91,208	$60,882
P + R + NP + RN	97	3.67	3.08	$91,208	$78,426
P + R + RN + CM + SW	75	3.67	2.25	$91,208	$64,432
P + R + RN + CM	192	3.67	3.21	$91,208	$77,719
P + R + RN + SW	37	3.67	2.96	$91,208	$90,085
P + R + RN	162	2.6	3.29	$73,448	$90,930
P + NP + RN + CM + SW	99	3.67	3.67	$91,208	$0
P + NP + RN + CM	114	3.67	1.88	$91,208	$90,816
P + NP + RN + SW	81	3.67	2.08	$91,208	$70,586
P + NP + RN	170	3.67	2.83	$91,208	$84,417
P + RN + CM + SW	140	3.67	3.13	$91,208	$91,208
P + RN + CM	314	3.67	2.92	$91,208	$90,741
P + RN + SW	87	3.67	3.17	$91,208	$89,721
P + RN	389	2.21	3.54	$91,208	$91,112
R + NP + RN + CM + SW	29	3.67	1.21	$91,208	$44,164
R + NP + RN + CM	59	3.67	2.38	$91,208	$80,332
R + NP + RN + SW	33	3.67	2.42	$91,208	$88,298
R + NP + RN	69	3.58	3.54	$91,208	$91,064
R + RN + CM + SW	63	3.67	2.92	$91,208	$80,420
R + RN + CM	222	3.67	2.88	$91,208	$90,100
R + RN + SW	68	3.08	2.92	$60,089	$87,276
R + RN	225	2.08	3.25	$47,565	$90,787
NP + RN + CM + SW	29	3.67	0.13	$91,208	$39,784
NP + RN + CM	41	3.67	1.92	$91,208	$67,351
NP + RN + SW	32	3.67	2.25	$91,208	$83,086
NP + RN	88	3.67	3.46	$91,208	$88,620

*P, physician; R, resident; NP, nurse practitioner; RN, registered nurse; CM, care manager; SW, social worker.

### 3.2 Key findings for odds ratios in high length of stay

Overall, the only team configuration model found to be associated with high length of stay in all patients, and in subgroups when stratified by heart failure severity, was the R + RN configuration, in which they were less likely to have a high length of stay. Importantly, we found four additional team configurations where all patients, patients with the most severe cases, and patients with the least severe cases were less likely to have a high length of stay. These found configurations were P + R + NP + RN + CM + SW, P + R + RN, P + RN + CM + SW, and P + RN.

To further address potentially unknown and confounding variables, adjusted odds ratios in Tables 5 and 6 were stratified by heart failure phenotype using ejection fraction rates.

**Table 5:** Logistic regression odds ratios of patients having a high LOS by severity of CHF.

Variables	Model 1: All patients high LOS odds ratio (95%CI)	Model 2: HFrEF patients high LOS odds ratio (95%CI)	Model 3: HFmrEF high LOS0 odds ratio (95%CI)	Model 4: HFpEF high LOS odds ratio (95%CI)
Patient characteristics				
Median age	1.04 (0.87–1.25)	0.90 (0.63–1.28)	2.48^a^ (1.43–4.30)	0.97 (0.77–1.23)
Male	1.013 (0.85–1.21)	0.64^a^ (0.45–0.92)	1.60 (0.94–2.72)	1.12 (0.89–1.41)
Caucasian American	0.85 (0.42–1.72)	0.99 (0.27–3.69)	0.41 (0.04–3.98)	0.75 (0.29–1.91)
African American	0.53 (0.26–1.09)	0.49 (0.13–1.87)	0.33 (0.03–3.26)	0.49 (0.19–1.28)
Asian American	0.59 (0.15–2.41)	1002748839 (0–∞)	0.36 (0–30.12)	0.34 (0.06–1.85)
American Indian/Alaskan Native	0.29 (0.05–1.78)	2203177504 (0–∞)	—	0.05^a^ (0–0.73)
Native Hawaiian/Other Pacific Islander	3.03078318 (0–∞)	2203177504 (0–∞)	—	—
Other Race	—	—	—	—
Hispanic/Latin/Spanish	0.42 (0.17–1.01)	0.43 (0.07–2.56)	0.09 (0–2.40)	0.42 (0.14–1.31)
Overall Health Severity: Van Walraven Elixhauser Comorbidity Score (VWECS)				
Low Mortality Risk (VWECS of −19 to 7)	—	0.11^a^ (0.04–0.31)	0.15^a^ (0.04–0.57)	—
Medium Mortality Risk (VWECS of 8–34)	3.05^a^ (2.43–3.82)	0.41 (0.16–1.09)	0.31 (0.09–1.10)	—
High Mortality Risk (VWECS of 35–61)	5.71^a^ (3.65–8.91)	—	—	—
Very High Mortality Risk (VWECS of 62–89)	9.6617344738 (0–∞)	—	—	—
Care team configuration				
P + R + NP + RN + CM + SW	7.32^a^ (1.62–33.03)	12.41^a^ (1.32–116.97)	1,684,925,686 (0–∞)	16.97^a^ (2.11–136.78)
P + R + NP + RN + CM	2.35 (0.91–6.06)	4.76 (0.76–29.84)	1.44 (0.15–13.87)	8.45^a^ (2.28–31.37)
P + R + NP + RN + SW	2.21 (0.81–6.01)	5.78 (0.96–34.81)	1223914370 (0–∞)	4.46^a^ (1.34–14.86)
P + R + NP + RN	0.55 (0.28–1.07)	1.08 (0.28–4.14)	2.17 (0.34–13.97)	1.08 (0.49–2.37)
P + R + RN + CM + SW	2.64^a^ (1.03–6.78)	4.52 (0.94–21.70)	1452658376 (0–∞)	6.02^a^ (1.75–20.69)
P + R + RN + CM	0.81 (0.44–1.50)	1.80 (0.56–5.73)	4.78^a^ (1.11–20.57)	1.52 (0.72–3.20)
P + R + RN + SW	0.71 (0.30–1.73)	6.95 (0.66–72.74)	0.49 (0.03–8.36)	1.41 (0.50–4)
P + R + RN	0.15^a^ (0.08–0.27)	0.28^a^ (0.09–0.86)	0.59 (0.12–2.76)	0.32^a^ (0.16–0.66)
P + NP + RN + CM + SW	6.13^a^ (1.98–18.95)	879,671,142 (0–∞)	2.39 (0.29–19.69)	18.08^a^ (3.91–83.62)
P + NP + RN + CM	1.47 (0.71–3.04)	2.75 (0.71–10.59)	7.27^a^ (1.04–51.03)	3.28^a^ (1.33–8.09)
P + NP + RN + SW	1.05 (0.49–2.24)	1.14 (0.31–4.23)	6.04 (0.46–78.44)	2.98^a^ (1.13–7.86)
P + NP + RN	0.48^a^ (0.26–0.88)	0.91 (0.31–2.71)	1.92 (0.37–10)	1.12 (0.54–2.32)
P + RN + CM + SW	2.25^a^ (1.08–4.71)	5.76^a^ (1.41–23.58)	3.88 (0.67–22.65)	5.80^a^ (2.26–14.89)
P + RN + CM	0.51^a^ (0.29–0.90)	1.05 (0.38–2.93)	1.54 (0.39–6.09)	1.20 (0.62–2.34)
P + RN + SW	0.38^a^ (0.19–0.74)	1.41 (0.38–5.20)	1.54 (0.29–8.11)	0.64 (0.28–1.44)
P + RN	0.11^a^ (0.06–0.19)	0.20^a^ (0.70–0.57)	0.53 (0.13–2.20)	0.23^a^ (0.12–0.44)
R + NP + RN + CM + SW	3.63 (0.78–16.91)	799,472,984 (0–∞)	985,138,249 (0–∞)	4.63 (0.92–23.23)
R + NP + RN + CM	0.68 (0.31–1.49)	1.56 (0.34–7.18)	4.98 (0.42–59.05)	1.32 (0.52–3.34)
R + NP + RN + SW	—	0.64 (0.13–3.21)	2.75 (0.12–63.83)	5.15^a^ (1.06–25.12)
R + NP + RN	0.23^a^ (0.12–0.47)	0.56 (0.17–1.88)	0.33 (0.03–4.32)	0.47 (0.20–1.14)
R + RN + CM + SW	1.43 (0.61–3.39)	2.18 (0.50–9.45)	3.10 (0.44–21.56)	5.46^a^ (1.43–20.85)
R + RN + CM	0.39^a^ (0.22–0.69)	0.74 (0.25–2.16)	2.11 (0.47–9.45)	0.79 (0.40–1.54)
R + RN + SW	0.19^a^ (0.09–0.39)	0.32 (0.08–1.19)	1.27 (0.14–11.97)	0.39^a^ (0.17–0.93)
R + RN	0.09^a^ (0.05–0.16)	0.19^a^ (0.06–0.58)	0.14^a^ (0.03–0.67)	0.21^a^ (0.11–0.43)
NP + RN + CM + SW	7.32 (0.93–57.75)	900,011,151 (0–∞)	2546140178 (0–∞)	10.22^a^ (1.23–84.93)
NP + RN + CM	—	2.45 (0.25–24.27)	1241993858 (0–∞)	3.70 (0.95–14.39)
NP + RN + SW	—	0.54 (0.11–2.64)	895,771,908 (0–∞)	2.07 (0.59–7.31)
NP + RN	0.42^a^ (0.21–0.83)	—	—	—

An “^a^” indicates a significant result with a *p*–value of less than 0.05. An “—” indicates a sample size too small to test for significance. Model 1 explained 35.1% of the variance in high LOS and correctly classified 75.3% of hospitalizations. Model 2 explained 38.4% of the variance in high LOS and correctly classified 76.8% of hospitalizations. Model 3 explained 41% of the variance in high LOS and correctly classified 72.9% of hospitalizations. Model 4 explained 36.9% of the variance in high LOS and correctly classified 75.3% of hospitalizations. “LOS” refers to length of stay.

**Table 6:** Logistic regression odds ratios of patients having a high-charge hospitalization by severity of CHF.

Variables	Model 1: All patients high-charge hospitalization odds ratio (95%CI)	Model 2: HFrEF patients high-charge hospitalization odds ratio (95%CI)	Model 3: HFmrEF patients high-charge hospitalization odds ratio (95%CI)	Model 4: HFpEF patients high-charge hospitalization odds ratio (95%CI)
Patient characteristics				
Median age	1.086 (0.90–1.30)	0.89 (0.62–1.26)	2.26^a^ (1.30–3.92)	1.04 (0.82–1.31)
Male	1.13 (0.95–1.35)	0.82 (0.58–1.17)	1.39 (0.82–2.36)	1.28^a^ (1.01–1.61)
Caucasian American	1.45 (0.74–2.85)	3.49 (1–12.78)	0.71 (0.07–7.65)	1.03 (0.42–2.54)
African American	0.75 (0.38–1.49)	1.54 (0.44–5.45)	0.43 (0.04–4.67)	0.53 (0.21–1.32)
Asian American	1.45 (0.35–5.96)	3223379333 (0–∞)	—	0.88 (0.16–4.88)
American Indian/Alaskan Native	1.14 (0.18–7.48)	7655177463 (0–∞)	—	0.32 (0.04–2.89)
Native Hawaiian/Other Pacific Islander	—	—	—	—
Other Race	—	—	—	—
Hispanic/Latin/Spanish	0.85 (0.36–2.01)	0.64 (0.11–3.76)	0.73 (0.04–15.19)	0.75 (0.25–2.28)
Overall Health Severity: Van Walraven Elixhauser Comorbidity Score (VWECS)				
Low Mortality Risk (VWECS of −19 to 7)	—	0.08^a^ (0.03–0.25)	0.07^a^ (0.02–0.37)	—
Medium Mortality Risk (VWECS of 8–34)	2.91^a^ (2.34–3.61)	0.25^a^ (0.08–0.76)	0.17^a^ (0.04–0.78)	—
High Mortality Risk (VWECS of 35–61)	10.59^a^ (6.22–18.03)	—	—	—
Very High Mortality Risk (VWECS of 62–89)	795,534,279 (0–∞)	—	—	—
Care team configuration				
P + R + NP + RN + CM + SW	7.72^a^ (2.19–27.13)	24.82^a^ (2.65–232)	1,719,244,108 (0–∞)	6.53^a^ (1.35–31.64)
P + R + NP + RN + CM	2.41^a^ (1.05–5.53)	5.62^a^ (1.05–30)	1.61 (0.17–15.51)	3.62^a^ (1.18–11.12)
P + R + NP + RN + SW	2.16 (0.91–5.12)	6.10^a^ (1.25–29.92)	1.09 (0.06–19.55)	2.74 (0.87–8.59)
P + R + NP + RN	1.13 (0.59–2.17)	1.69 (0.46–6.22)	2.57 (0.39–16.91)	1.37 (0.59–3.22)
P + R + RN + CM + SW	2.22^a^ (1.02–4.85)	6.64^a^ (1.54–28.66)	7.37 (0.64–84.38)	2.28 (0.80–6.51)
P + R + RN + CM	1.77 (0.89–3.20)	3.67^a^ (1.16–11.63)	6.82^a^ (1.49–31.21)	1.86 (0.83–4.17)
P + R + RN + SW	0.51 (0.22–1.16)	1.47 (0.26–8.23)	2.76 (0.15–50.85)	0.50 (0.18–1.39)
P + R + RN	0.43^a^ (0.24–0.75)	1.14 (0.38–3.41)	1.66 (0.35–7.83)	0.40^a^ (0.19–0.84)
P + NP + RN + CM + SW	644,113,053 (0–∞)	1689002728 (0–∞)	5222414211922100 (0–∞)	725,897,639 (0–∞)
P + NP + RN + CM	2.70^a^ (1.32–5.52)	7.42^a^ (1.79–30.81)	17.78^a^ (1.61–196.45)	2.53 (1–6.39)
P + NP + RN + SW	1.93 (0.91–4.10)	2.64 (0.70–9.94)	6.83 (0.50–92.68)	2.71 (0.97–7.62)
P + NP + RN	0.79 (0.45–1.38)	1.24 (0.43–3.57)	2.91 (0.52–16.17)	1.06 (0.49–2.31)
P + RN + CM + SW	3.12^a^ (1.57–6.17)	10.73^a^ (2.67–43.17)	3.56 (0.61–20.76)	3.58^a^ (1.43–8.94)
P + RN + CM	1.43 (0.85–2.42)	3.29^a^ (1.18–9.18)	3.82 (0.91–16.01)	1.59 (0.78–3.27)
P + RN + SW	0.87 (0.46–1.67)	3.39 (0.91–12.68)	0.91 (0.17–4.82)	1.04 (0.43–2.49)
P + RN	0.48^a^ (0.29–0.79)	1.09 (0.40–2.97)	1.30 (0.31–5.48)	0.53 (0.28–1.04)
R + NP + RN + CM + SW	2.64 (0.82–8.49)	3.01 (0.48–19.11)	1191989554 (0–∞)	3.81 (0.74–19.50)
R + NP + RN + CM	0.76 (0.37–1.57)	3.22 (0.70–14.86)	1.96 (0.23–16.59)	0.71 (0.28–1.80)
R + NP + RN + SW	—	2.11 (0.39–11.26)	2.79 (0.11–69.85)	1.35 (0.41–4.48)
R + NP + RN	0.39^a^ (0.20–0.76)	0.80 (0.24–2.61)	0.35 (0.03–4.61)	0.50 (0.20–1.26)
R + RN + CM + SW	1.64 (0.75–3.58)	2.42 (0.63–9.29)	3.68 (0.51–26.56)	3.12 (0.90–10.75)
R + RN + CM	0.66 (0.39–1.11)	2.01 (0.69–5.84)	2.36 (0.51–10.87)	0.60 (0.30–1.21)
R + RN + SW	0.19^a^ (0.09–0.37)	0.53 (0.14–2.01)	0.20 (0.02–2.62)	0.19^a^ (0.08–0.47)
R + RN	0.11^a^ (0.06–0.20)	0.28^a^ (0.09–0.88)	0.33 (0.07–1.51)	0.11^a^ (0.05–0.24)
R + RN	0.11^a^ (0.06–0.20)	0.28^a^ (0.09–0.88)	0.33 (0.07–1.51)	0.11^a^ (0.05–0.24)
NP + RN + CM + SW	3.61 (0.10–13.10)	1727004469 (0–∞)	4.66 (0.34–64.68)	3.66 (0.73–18.40
NP + RN + CM	—	936,463,463 (0–∞)	1.25 (0.14–11.38)	0.78 (0.27–2.24)
NP + RN + SW	—	1.66 (0.32–8.63)	880,980,672 (0–∞)	1.23 (0.36–4.16)
NP + RN	0.65 (0.34–1.24)	—	—	—

An “^a^” indicates a significant result with a *p*-value of less than 0.05. An “—” indicates a sample size too small to test for significance. Model 1 explained 31.7% of the variance in high-charge hospitalizations and correctly classified 74.4% of hospitalizations. Model 2 explained 34.6% of the variance in high-charge hospitalizations and correctly classified 75% of hospitalizations. Model 3 explained 34.6% of the variance in high-charge hospitalizations and correctly classified 75.3% of hospitalizations. Model 4 explained 33.7% of the variance in high-charge hospitalizations and correctly classified 75.5% of hospitalizations.

### 3.3 Key findings for odds ratios in high-charge hospitalizations

Overall, there were no team configuration models found to be associated with a high-charge hospitalization in all patients, and in all subgroups when stratified by heart failure severity. However, importantly, we found four team configurations where all patients, patients with the most severe cases, and patients with the least severe cases were less likely to have a high-charger hospitalization. These four configurations were P + R + NP + RN + CM + SW, P + R + NP + RN + CM, P + RN + CM + SW, and R + RN.

## 4 Discussion

### 4.1 Summary of key findings

Registered nurses were the only care role found within all team configurations. Broadly, patients with larger team configurations (i.e., with four or more different types of care roles) had higher length of stay and charges than smaller team configurations (i.e., with two to three different types of care roles). Importantly, we found that the only team configuration associated with high length of stay in all patients and in subgroups when stratified by heart failure severity, was the R + RN configuration, in which they were less likely to have a high length of stay. Notably, there were no team configuration models found to be associated with high charge hospitalizations in all patients and in all subgroups when stratified by heart failure severity. We found three team configurations where all patients, patients with the most severe cases, and patients with the least severe cases were less likely to have a high length of stay and high charge hospitalization. These three configurations were P + R + NP + RN + CM + SW, P + RN + CM + SW, and R + RN. The study identified several individual care team configurations that were associated with either high length of stay or high-charges in patients with CHF.

Our findings provide a foundation for satisfying calls from the Joint Committee of the American College of Cardiology and American Heart Association to investigate the role of care team configuration in reducing hospitalizations and related charges. Our findings establish an evidence base that indicates that care team configurations are related to length of stay and charges. Based on these findings, we now recommend that smaller care team configurations, primarily the R + RN team configuration (i.e., a resident and a registered nurse), be tested within experimental designs as the standard of care for use in patients with CHF. Additionally, we recommend that registered nurses lead the integration and use of the VWECS into the electronic health record at the point of care. Evidence supporting these recommendations are discussed below.

### 4.2 Implications for advancing inpatient care in CHF

Prior research has demonstrated that both patients with the severest cases of CHF and patients with significant comorbidity have a larger combination of care roles on their care teams than patients with other chronic conditions (3, 8, 14). CHF patient care team configurations generally include an array of general practitioners with broad care expertise and those with specific expertise, credentials, and/or certifications in CHF (25). Notably, team configurations that all shared and included a nurse practitioner, registered nurse, care manager, and social worker had the highest charges and length of stay than configurations that did not collectively include all of these roles.

Notably, the presence of social workers and care managers among team configurations had a similar association with outcomes. Both social workers and care managers were found in 50% (i.e., 14 of the 28 team configurations). Eleven of these 14 models had higher median length of stay and charges. Of the 11 team configurations, 10 were large team configurations (i.e., composed of four or more different types of care roles). Furthermore, large care teams that included either a social worker or a care manager had patients with greater odds of having a high-charge hospitalization and a high length of stay in both the sickest patients with CHF (i.e., HFrEF) and the healthiest patients with CHF (i.e., HFpEF). The social workers' association with high-charge hospitalizations and high length of stay reinforces their role of supporting the financial needs of patients with CHF (e.g., transportation, medications, and translating discount coupons with pharmacists) because social workers have traditionally been engaged in the care of patients with the greatest financial needs, which are driven by poorer states of health and the most consistent need for care (10). The care manager's association with high-charge hospitalizations and high length of stay within these large team configurations reinforces their role of assisting with the discharge planning of patients with CHF because adherence to treatment plans, including post-discharge visit follow-up within 7–14 days, is an active strategy for minimizing the incurrence of potential charges and the length of future hospital stays (10). These findings motivate the need for additional inquiry regarding the specific financial support and discharge planning tasks performed by social workers and care managers working within large multidisciplinary care teams, and how they are associated with charges and length of stay. To advance inquiry, formal task analysis approaches may be an effective method of understanding how differences in the specific tasks performed by social workers and care managers are associated with outcomes because of the approaches' ability to isolate and classify the tasks performed by each role.

Registered nurses were the only specific care team role that was found within all care team configurations. This was consistent with the prior research demonstrating the vast and highly engaged role of the registered nurse in high-quality CHF care delivery (e.g., triage, ongoing monitoring, discharge planning, and end-of-life care) (8, 14, 26). Furthermore, a recent study of 5,962 patients with CHF found that those with a registered nurse providing care delivery were 88% less likely to have a hospitalization and 50% less likely to have a high number of readmissions over a seven-year period (14). Based on current and previous findings, increasing the number of registered nurses on the care teams would likely improve hospitalization rates and length of stay. However, this would as adversely increase the charges for care. This would also inflame the global challenge hospitals have of managing the three elements of the “Iron Triangle” of healthcare (i.e., access, quality, and cost), where improving one element causes a negative impact on one of the other two elements (27). Resolving this challenge has been *a priori*ty of the World Health Organization since 1948. A recommendation from the World Health Organization's for addressing cost containment is for hospitals to train and utilize auxiliary nursing personnel such as licensed practical nurses (28). To do so, additional inquiry is needed to identify specific care tasks that are currently being performed by registered nurses that are drivers of cost. These care tasks would likely include tasks that are time consuming for registered nurses and those tasks which require a significant amount of technical knowledge or specialized expertise. Once identified, these tasks could be incorporated into formal training programs of auxiliary nursing personnel.

These findings indicate that registered nurses will play a critical role in any future reconfiguration of the care team. However, we did not test a team configuration without a registered nurse because none were available. Additional inquiries that employ experimental designs are needed to isolate and explore causation. Yet, our findings demonstrate that the highly engaged role of registered nurses provides a significant opportunity for increased registered nurse-led solutions for reducing length of stay and charges in patients with CHF, as the American College of Cardiology and American Heart Association have called for care team reconfiguration (10–13). In the context of current literature, our findings indicate that registered nurses may be particularly effective at advancing attempts to improve and integrate real-time risk stratification approaches at the point of care. For example, a nurse-led, randomized trial of a multidisciplinary care team configuration reduced length of stay associated with readmission by 56.2% in a study of 282 patients with CHF (29). Currently, the most salient method of risk stratification is the VWECS, which was inadvertently and further validated by this study. Our study found that the quartiles of the VWECS were statistically significantly associated with length of stay and charges. More specifically, this study found that patients with CHF with a lower VWECS (i.e., lower mortality risk) were less likely to have a high length of stay and a high-charge hospitalization. This suggests that the VWECS and its quartiles are an indicator of care demand because the VWECS represents the level of severity of a patient's morbidity. For example, in the severest CHF cases (i.e., HFrEF patients), those with a VWECS of −19 to 7 (i.e., low mortality risk) were 89% less likely to have a high length of stay and 92% less likely to have a high-charge hospitalization, which could represent a low demand for care.

Unfortunately, the VWECS is not readily available in the electronic health record systems of hospitals within the United States for real-time use in care (24). The use of the VWECS has been limited to research efforts focused on retrospectively evaluating the severity of a patient's health. With these results and an expanse of prior validations (20, 24, 30, 31), the VWECS should now be integrated into real-time risk stratification of patients with CHF during care delivery. Not only did this study further validate the application of the VWECS in research and clinical care, but it also inadvertently validated our novel statistical approach of segmenting the total VWECS (i.e., ranging from −19 to 89) into four equal quartiles (i.e., −19 to 7; 8–34; 35–61; 62–89) to reflect low to very high likelihood of a CHF patient's mortality risk. Therefore, to advance the implementation of the VWECS into care delivery, further assessments must be conducted to identify the specific caring and care tasks performed by registered nurses that are associated with improved mortality, length of stay, and charges, at each level of the four segmented quartiles of the VWECS. For example, current clinical practice guidelines recommend that registered nurses perform a two to three grams/day sodium restriction intervention in thosewith ejection fraction rates of less than 40% (3). This level of guidance and specificity is provided for the segmented ejection fraction rates/levels (i.e., “HFrEF”; ≤40%, “HFmrEF”; ≥41%–49%, “HFpEF”; ≥50%), specifically reflecting heart failure severity. However, the same level of guidance and specificity has not been provided within the VWECS nor within its four segmented quartiles, which could substantially support care and clinical decision-making if each level is associated with a specific set of registered nurse-led care tasks. This advancement is critical to the survival of patients with CHF because it is estimated that more than 85% have two or more additional chronic conditions (e.g., anemia, hypertension, ischemic heart disease, diabetes) which are factored into the VWECS by weighting the seriousness of each condition (3, 20, 24). Yet, the VWECS is not advocated for or discussed within the 2022 Joint Committee of the American College of Cardiology and American Heart Association's guidelines for the management of heart failure. With the registered nurse so heavily associated with positive outcomes, the role could be expanded to retrieve the VWECS from their hospital's electronic health record within the scope of their initial triage and assessment of a CHF patient. For resource-constrained hospital environments with less comprehensive electronic health record systems, the VWECS can be calculated manually by (1) initially identifying a diagnosis of the VWECS's 21 chronic conditions, (2) summing the numerical weight associated with each of the conditions that are present within a patient, and (3) identifying the quartile associated with the patient's total score.

### 4.3 Implications for team configuration in CHF care

Generally, larger team configurations (i.e., with four or more different types of care roles) had a higher median charge per care hospitalization and higher median length of stay than smaller care team configurations (i.e., with two and three different types of care roles). There were only a few exceptions in charges (i.e., “P + R + RN + SW”, “NP + RN + CM”, and “P + RN + CM”) and length of stay (i.e., “NP + RN + SW” and “NP + RN + CM”). Correspondingly, of those statistically significant team configurations, a larger number of care roles providing care during an hospitalization was associated with a greater likelihood of a patient having a high length of stay hospitalization and a high-charge hospitalization among all patients and when stratified by heart failure severity. For example, the “P + R + NP + RN + CM + SW” team configuration had the greatest likelihood of patients having a high length of stay and high-charge hospitalization, 7.32 and 7.72 times more likely, in all patients. In HFrEF patients (i.e., the most severe CHF cases), 12.41 and 24.82 times more likely to have a high length of stay and high-charge hospitalization, respectively. These findings were not consistent with prior studies on the effectiveness of multidisciplinary care teams being associated with improved care outcomes. For example, a large multidisciplinary care team configuration which included a physician, case manager, pharmacist, social worker, and a dietitian, was found to reduce length of stay from 5.7 days to 5 days and 30-day readmissions decreased from 27.6% to 17.22% in 181 patients with CHF (32). Twenty-nine additional trials of multidisciplinary team configurations have found positive results that include a 25% reduction in mortality risk, a 26% reduction in CHF hospitalizations, and a 19% reduction in hospitalizations (33). Correspondingly, there have been many calls for increasing the number and types of multidisciplinary team configuration interventions in CHF care (33, 34). These findings reflect the need to expand the responsibilities of hospitalized patients with CHF beyond the responsibility of the cardiologists. Recent qualitative studies have highlighted the voices of cardiologists who have advocated for having multidisciplinary team configurations to help carry the workload of providing such highly intensive care (25). More specifically, cardiologists believe that multidisciplinary team configurations can advance CHF care by expanding the role of the registered nurse and improving electronic health record data (25), both of which are consistent with our recommendation to have registered nurses lead the integration and use of the VWECS into the electronic health record at the point-of-care.

Furthermore, significant care team configurations that included both a care manager and a social worker were associated with a greater likelihood of a patient having a high length of stay hospitalization in all patients and when stratified by heart failure severity. For example, the “P + RN + CM + SW” was 2.25, 5.76, and 5.80 times more likely to have a high length of stay in all patients, HFrEF patients, and HFpEF patients, respectively. A recent study of 54,664 patients with multiple chronic conditions, including CHF, found that any combination of two or more physicians, residents, nurse practitioners, registered nurses, or care managers providing care during a care delivery hospitalization was associated with a 46%–98% decreased likelihood of having a high number of hospitalizations (i.e., with a length of stay of ≥0 days) over a seven-year period (8). Similarly, any combination of two or more residents and/or registered nurses was associated with an 11%–13% increased odds of having a high-charge hospitalization (8). These results indicated that care team configuration data within the electronic health record systems of hospitals could be an effective method of isolating and tracking high-risk patients, as high length of stay and high-charge hospitalizations are significant indicators of heavy utilization of health care systems. This will be particularly impactful to population health management in rural, small, and resource-constrained hospitals throughout geographical areas with a high prevalence of CHF. For example, this study's sample of 3,099 patients with CHF were from Arkansas, which has the third highest concentration of CHF death rates (i.e., 258.9–563 per 100,000) of all 50 states and where many of its counties have CHF prevalence and mortality risk that are nearly twice the national average (35–37).

While the results did not determine that specific care team configurations caused high length of stay or high-charge hospitalizations, the results demonstrated that many care team configurations were associated with high length of stay and high-charge hospitalizations. Therefore, a recommendation for future research is to evaluate the causes and effectiveness of specific team configurations using comparative, experimental, and mixed methods designs. Future research that employs comparative designs should approach these designs by using categorical variables and setting a reference level for the analysis as opposed to the approach of using all dichotomous variables that were employed within this design. This would support multi-level comparisons of many of the variables. For example, heart failure severity contained three binary variables (i.e., HFrEF, HFmrEF, and HFpEF) that could be treated as categories to compare each level with the reference instead of each level against all other levels as we did (i.e., patients with HFrEF vs. patients without HFrEF). The team configurations that should be the focus of future research are those that were associated with low length of stay and low charge hospitalizations, which were generally smaller team configurations (i.e., with two and three different types of care roles) in the worst cases of CHF (i.e., HFrEF). These include the following: P + R + RN, P + RN, and R + RN. Future studies should also examine other integral areas of the domain including how the tasks of the teams are organized and the influence of communication and interactions among the teams, and patients, as care is delivered.

Furthermore, it is universally known that many covariates (e.g., specific shifts, days of the week, time of year) can influence the length of stay and charges of patients and the configuration of their care teams through clinic staffing and care team availability. Here, the study's approach to evaluating all possible care team configurations limited the ability of the regression models to account for all salient covariates without losing significance. However, the findings presented here narrow the scope of inquiry for future research on care team configuration. Future research should incorporate these covariates into smaller regression models that only include the significant configurations that were identified here and the covariates, excluding all non-significant team configurations (*p* > 0.05).

### 4.4 Limitations

The study did not evaluate all possible combinations and permutations of care team configurations that are potentially found within the care hospitalizations of hospitalized patients with CHF. The analysis was limited by the inclusion and exclusion criteria for patients and clinicians as well as the fact that not all possible configurations were found within the data provided by the Arkansas Clinical Data Repository. Notably, the work performed by registered nurses is often supplemented by auxiliary nursing personnel such as licensed practical nurses. However, auxiliary nursing personnel provided care in less than one percent of the sample and did not provide an adequate sample size for identifying significant associations with length of stay and charges.

The sample size (3,099) was much smaller than the population of total encounters available (49,100). Yet, representative was maintained and the potential for bias was reduced because randomization was applied in selecting the hospitalizations that were analyzed and there were no systematic or random errors identified in the data. The sample was smaller because of the statistical assumption of the logistic regression which required independence of observation and limited the analysis to only one hospitalization per patient. Analyzing more than one hospitalization per patient would have violated this assumption and have been a new, more advanced study (i.e., understanding how length of stay and charges changed across different team configurations for a patient over time). However, a foundation needed to be established through the current, basic research question of how length of stay and charges were associated with team configurations because this was unknown. Now that this association is known, future studies can pursue more advanced questions including how LOS and charges change when a patient has a different care team configuration.

We did not consistently find that the two highest quartiles of the VWECS (i.e., high mortality risk with scores of 35–61 which accounted for 6% of patients, and very high mortality risk with scores of 62–89 which accounted for less than 1% of patients) were significantly associated with high length of stay or a high-charge hospitalization. We believe this was limited by, and due to the small sample of patients with these scores that fell between these two quartiles (i.e., a small sample of patients with poor overall health based solely on comorbidities).

Additionally, hospitals sometimes charge more for the same service depending on the insurance (or lack thereof) which could have affected the charges billed to payors. However, patient selection was not influenced by charge categorizations such as diagnostic-related groups. Our random selection of patient hospitalizations reduced the likelihood of any potential systematic errors which could have impacted the number of charges billed to payors. Furthermore, this analysis did not include, distinguish, or isolate specialty clinicians with specific expertise in CHF (cardiologists, CHF nurses) from those with broad expertise in the analysis because (1) it is well established that these specialists are associated with positive patient outcomes and (2) the Arkansas Clinical Data Repository data did not include those specialty roles. Finally, the Arkansas Clinical Data Repository data was complete and correct to the best of our knowledge and the knowledge of the Arkansas Clinical Data Repository. However, we did not have direct access to patient records within the electronic health record to feasibly control for the correctness of the data. Ideally, controlling for correctness would have been performed by conducting a chart review in the electronic health record, comparing a representative sample of the data received from the Arkansas Clinical Data Repository to the data in the charts of the electronic health records of patients.

## 5 Conclusions

Specific combinations of care roles that provide care to hospitalized patients with CHF are associated with a high length of stay and a high-charge hospitalization. Care team configuration data within electronic health record systems of hospitals could be an effective method of isolating and tracking high-risk patients. Within multidisciplinary care team configurations, registered nurses may be particularly effective in advancing real-time risk stratification approaches at the point of care. The integration of electronic health record-based risk scores and clinical assessments could reduce length of stay and charges in patients with CHF.

## Data Availability

The data analyzed in this study is subject to the following licenses/restrictions: the dataset presented in this article is not readily available because it contains information that could compromise the privacy of patients and their clinicians. Requests to access these datasets should be directed to the UAMS IRB, irb@uams.edu.
